# Time-dependent microbial colonisation of temporary external fixations: a prospective descriptive study

**DOI:** 10.1186/s13018-026-06815-2

**Published:** 2026-03-25

**Authors:** Thomas Kusnik, Ole Somberg, Thomas Rosteius, Christopher Ull, Martin Franz Hoffmann, Matthias Königshausen, Thomas Armin Schildhauer, Sebastian Lotzien

**Affiliations:** 1 Department of General and Trauma Surgery, BG University Hospital Bergmannsheil Bochum, Hospital of the Ruhr University Bochum, Bürkle-de-la-Camp Platz 1, 44789 Bochum, Germany; 2 Department of Orthopaedics and Trauma Surgery, St. Josefs Hospital, Wilhelm-Schmidt-Str. 44, 44263 Dortmund, Germany

**Keywords:** External fixation colonisation risk factors ASA score sonication

## Abstract

**Background:**

The temporary use of external fixators is an established method for stabilising fractures, particularly for injuries that do not initially allow definitive treatment. Despite its frequent use, it remains unclear whether the interval between fixator application and subsequent definitive fracture treatment affects microbial colonisation of the fixation screws prior to internal fixation.

**Methods:**

This prospective, single-centre study included patients aged 18 years or older between April 2023 and April 2024 who received a temporary fixator during initial fracture management. During definitive osteosynthesis, two bone-anchored Schanz screws were removed under sterile conditions and processed by sonication; the study was approved by the local ethics committee with written informed consent obtained from all participants. The primary endpoint was the incidence of positive sonication. In cases with microbial detection, the microbial spectrum was assessed, and the influence of fixation time and patient-related factors on colonisation and microbial distribution was analysed. Statistical analyses included chi-square testing, univariate analyses, and multivariable logistic regression with fixation duration modelled both dichotomously and as a continuous variable.

**Results:**

A total of 271 patients (*n* = 542 samples; two screw tips per patient processed together) were included. The median fixation time until definitive osteosynthesis was 8 days. Positive microbial colonisation was observed in 88 patients (32.5%). Patients with an external fixation time > 8 days (*n* = 120) showed a significantly higher colonisation rate of 43.3% (odds ratio 2.44, 95% CI 1.46–4.09; *p* = 0.001) compared with patients with ≤ 8 days (*n* = 151; colonisation rate 23.8%; *p* < 0.001). Frequently detected organisms included Staphylococcus epidermidis, Staphylococcus capitis and Staphylococcus aureus. Pre-existing conditions such as type 2 diabetes mellitus, nicotine abuse and BMI showed no significant impact on colonisation. An ASA score ≥ 3 was associated with a higher colonisation rate in multivariate analysis (OR 1.59; 95% CI 1.01–2.49; *p* = 0.043).

**Conclusions:**

Fixation duration showed a significant time-dependent association with microbiologically detected colonisation of Schanz screw tips. When modelled continuously, each additional day of fixation was associated with increased odds of colonisation. An ASA score ≥ 3 was independently associated with colonisation probability.

## Background

External fixation is pivotal in staged fracture care when soft-tissue damage or physiological instability delays definitive osteosynthesis. In polytrauma, it is a fundamental damage-control intervention that ensures provisional stability until conditions allow definitive management [1].

Despite advances in pre-hospital logistics and in-hospital resuscitation, the burden of severe trauma remains substantial globally. A recent systematic review of polytrauma patients with an Injury Severity Score > 15 (ISS) admitted to intensive care units found all-cause mortality around 14.4% in Europe, 22.6% in South America, and 9.6% in North America [2, 3]. In Germany alone approximately 31 000 severely injured patients are treated annually, with an acute in-hospital mortality of roughly 12% [4]. Although the incidence of complex injury patterns remains largely unchanged, shifts in injury mechanisms, including emerging mobility devices, demographic aging and high-energy leisure accidents, have increased the proportion of cases requiring staged, protocol-driven management pathways [5–8].

The timing of definitive internal fixation in staged fracture management is largely determined by the patient’s overall physiological condition and the status of the surrounding soft tissues. However, previous clinical studies have shown that, in open fractures, prolonged external fixator duration can be associated with an increased risk of fracture related infections [9, 10]. Aside from the initial contamination that can occur in open fractures, external fixation screws may act as conduits for pathogen transfer, creating a direct pathway from the external environment into the medullary canal [11]. With reported pin-site infection rates ranging from nearly 0% to 100%, there is concern that pin-related inoculation mechanisms may represent a microbiological source that warrants further investigation in relation to infection outcomes [12].

However, it remains unclear to what extent the duration of external fixation contributes to microbial colonisation of Schanz screws and how patient-related factors such as nicotine abuse, diabetes mellitus, obesity, and elevated ASA score (American Society of Anesthesiologists) modify this risk. Therefore, we conducted a laboratory-based study to investigate the relationship between fixator duration and microbiological colonisation of Schanz screws under uniform perioperative prophylaxis and standardised sonication-based diagnostics.

## Methods

This prospective single-centre clinical study was conducted over a 12-month period between 1 April 2023 and 1 April 2024 and was approved by the local institutional review board (Reg. Nr.: 2023-681-f-S). The primary objective was to analyse whether the duration of temporary external fixation affects the microbiological colonisation of Schanz screws, assessed by quantitative sonication. Secondary objectives included the description of the microbial spectrum and the evaluation of patient-dependent factors that may influence colonisation.

All adult patients (≥ 18 years) who underwent temporary external fixation as initial management of a closed fracture and subsequently underwent definitive open reduction and internal fixation of the affected limb in our clinic during the specified study period were eligible. Patients were excluded if they were younger than 18 years, did not provide informed consent, presented with any pre-existing infection at admission (e.g. urinary tract infection, pneumonia), received any form of ongoing antimicrobial therapy prior to the removal of the fixation, or sustained open fractures. Only closed injuries were analysed to avoid confounding environmental contamination and only patients who received the external fixation at our institution were included.

The sample size was determined using an a priori power analysis (G*Power v.3.1), assuming a two-sided comparison of two independent groups based on colonisation rates. With a total sample size of *n* = 271, effect size w = 0.21 and α = 0.05, the calculated statistical power was 0.94. Conversion time was analysed in two groups: ≤ 8 days (Group 1) and > 8 days (Group 2). These cut-off values were selected according to the median fixation time within the dataset and reflect the clinical routine of performing definitive osteosynthesis within the first week after initial stabilisation. Dichotomisation was an analytical decision to obtain balanced groups for hypothesis testing, not a biological threshold. In addition to dichotomized analyses, fixation duration was modelled as a continuous variable in multivariable logistic regression. To explore potential non-linearity, a quadratic term (duration^2^) was tested.

**Fig. 1:**
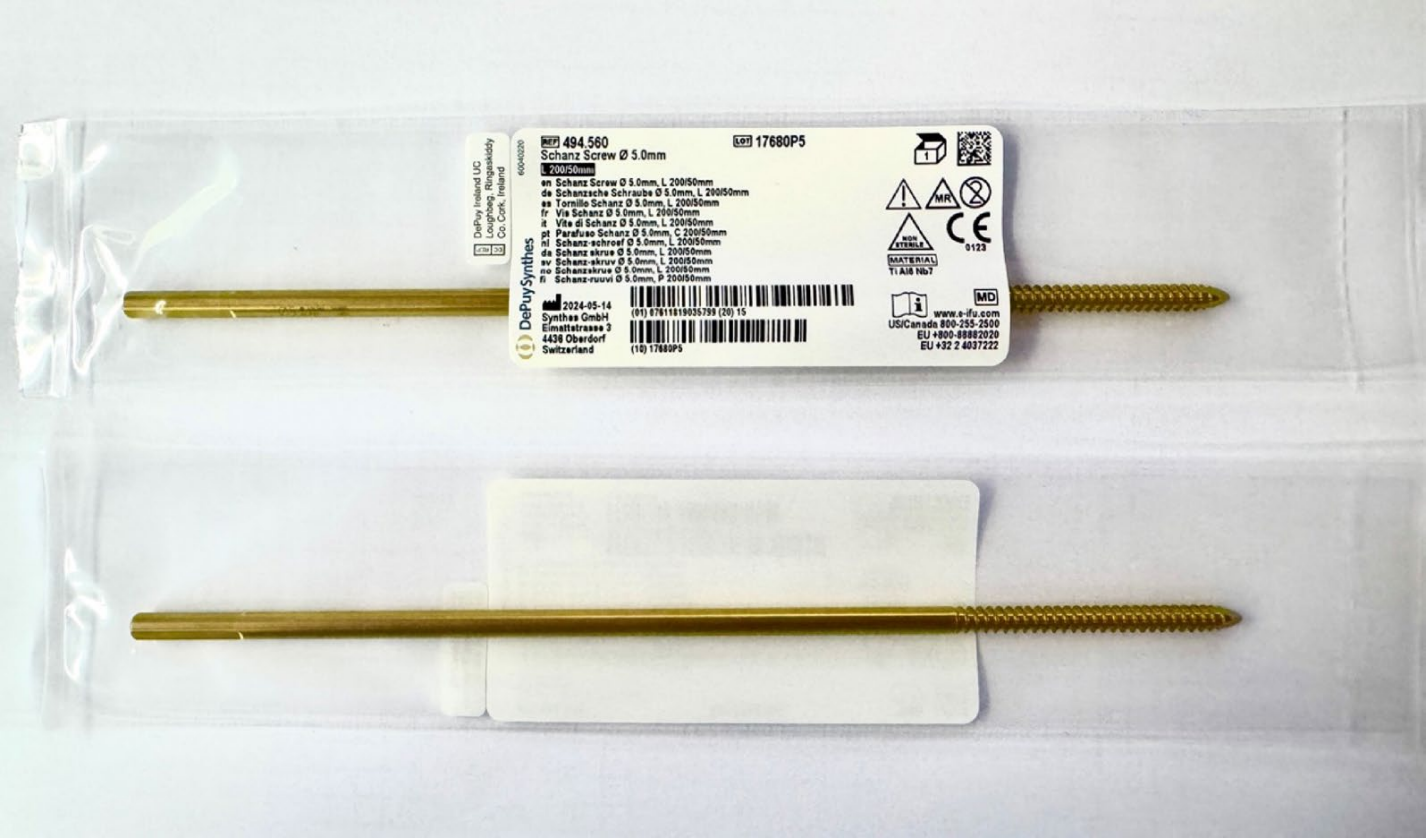
Typical Schanz screw used in the context of external fixation

Two Schanz screws were removed according to a predefined, standardised selection protocol. To account for potential local contamination, screws were selected both close to the fixation site within the area of possible fracture haematoma and distant from the fracture site. Screws showing clinical signs of pin-track irritation, loosening, soft-tissue compromise, or visible inflammation were systematically excluded. This approach ensured reproducible sampling conditions and comparable exposure of the pin tips across all patients. For consistency of material properties, all screws were identical titanium alloy (TiAl6Nb7) models from a single manufacturer (DePuy Synthes), measuring 5 mm in diameter and 200 mm in length (Fig. 1).

**Fig. 2:**
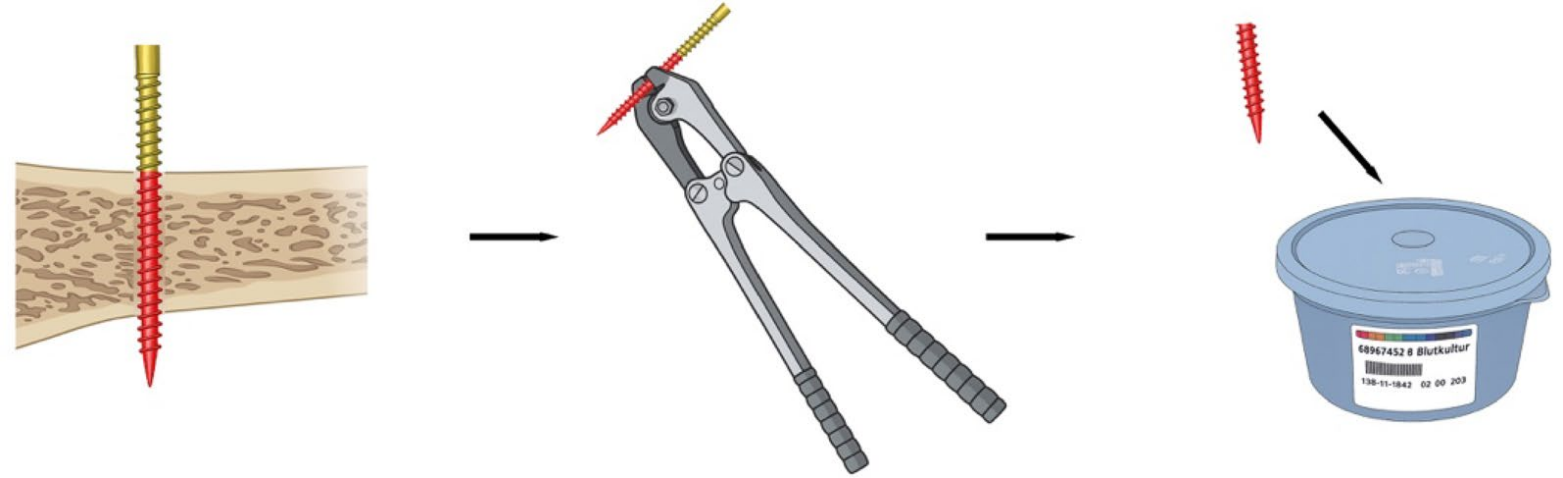
Sampling process. To collect the sample, the pin was grasped approximately 0.5–1 cm from the tip using sterile surgical forceps. The pin was then cut off directly at the forceps using sterilised wire cutters. The two extracted pin tips were placed together in a sterile sonication container and processed and analysed as a single sample under aseptic conditions

Removal of the fixation screws followed a uniform aseptic protocol. In the operating theatre, after the decision for definitive internal fixation was confirmed and the patient positioned, the external fixator was pre-cleaned using sterile compresses soaked in an alcohol-based disinfectant (Octeniderm, Schülke). Crusts or debris at the pin–skin interface were removed carefully without traumatising the surrounding tissue. The skin was disinfected three times with Octeniderm, observing manufacturer-specified exposure times. After complete drying, the screw was loosened proximally and removed without allowing the tip to contact the skin. The distal, intramedullary segment was grasped with sterile forceps and cut using sterilised wire cutters. The tip was immediately placed in a sterile low-volume polypropylene container. Containers were sealed and transported to the central microbiology laboratory within approximately 20 min (Fig. 2).

A positive sonication result was defined as ≥ 10³ colony-forming units per millilitre (CFU/ml).

Baseline patient characteristics were recorded from admission reports, medical history forms and preoperative anaesthesiology assessments. Documented variables included gender, height, weight, body mass index (BMI), ASA classification, diabetes mellitus, cardiovascular diseases, and active nicotine use. All data were acquired using standardised institutional documentation routines.

Sonication was performed using a low-frequency ultrasonic bath (BactoSonic; 40 kHz, 0.1–1 W/cm^2^). Before processing, each vessel was checked for integrity and completeness. The tips were moistened with sterile distilled water (Ampuwa, Fresenius Kabi). Samples were shaken for 30 s and then sonicated for 60 s under continuous cooling to avoid heating artefacts. Following sonication, the fluid was aspirated using a sterile syringe. Aliquots were inoculated onto agar plates using standardised spreading techniques to allow quantitative and qualitative isolation of microorganisms. Additional inoculation of aerobic and anaerobic blood culture bottles was performed.

**Fig. 3:**
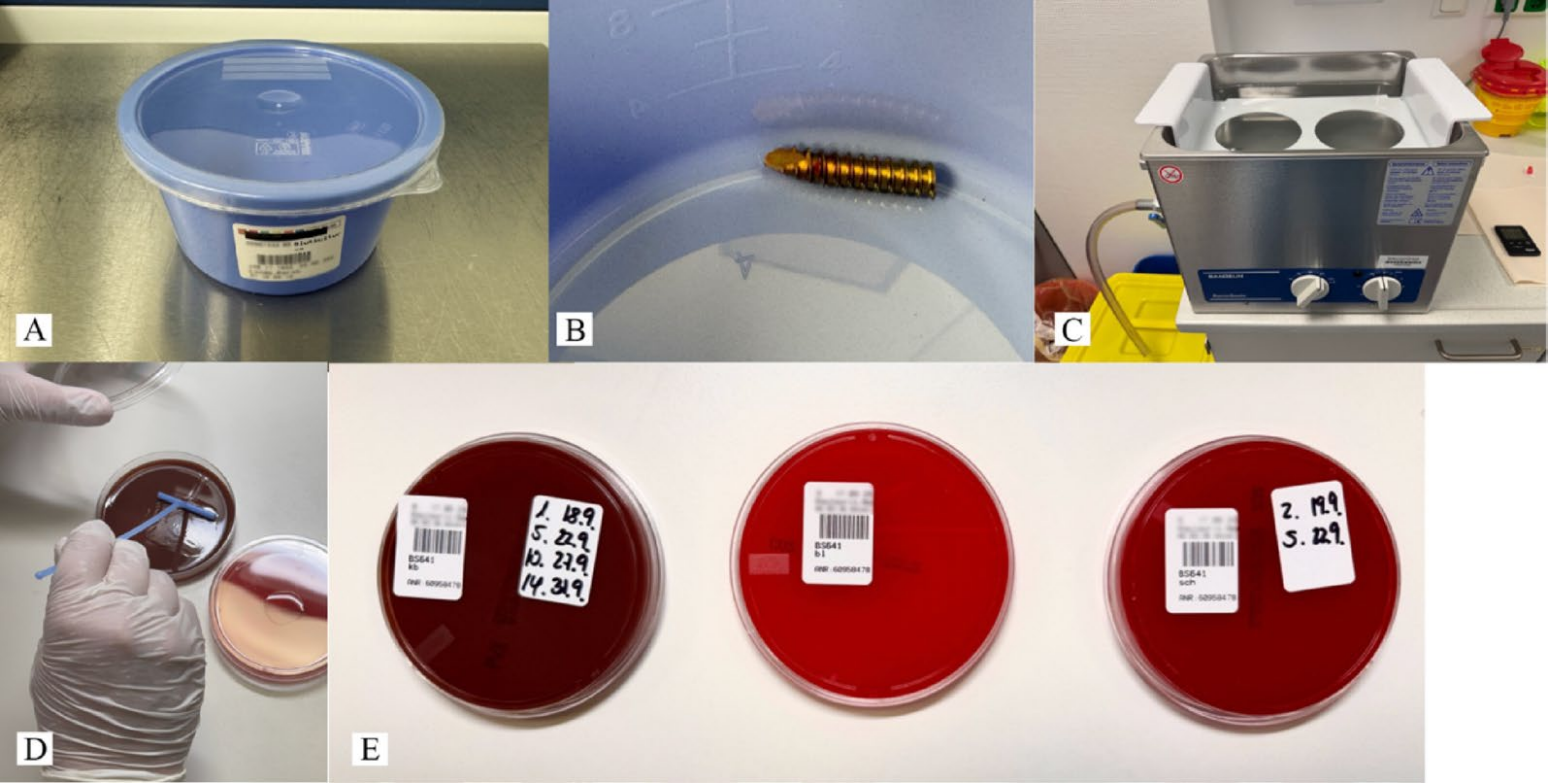
Illustration of the standardised microbiological processing procedure. Standardised container for storing the samples taken (**A**); pin tip removed in the sonication vessel (**B**); BactoSonic sonication device, Bandelin – the sonication vessel is placed in a corresponding holder and immersed in the ultrasonic bath (**C**); Spreading the sonication fluid obtained onto agar plates (**D**); Incubated agar plates after application of the sonication fluid with different culture media: cooked blood agar plate, blood agar plate and Schaedler agar plate (**E**)

Three different culture media were used: chocolate blood agar, blood agar, and Schaedler agar. Blood agar plates received 500 µl of sonication fluid and were incubated aerobically at 37 °C with evaluations on days 1, 5, 10 and 14. Schaedler plates were incubated anaerobically and evaluated on days 2 and 5. All cultures were incubated for a total of 14 days to ensure sufficient detection of slow-growing organisms. Culture processing followed validated institutional protocols (Fig. 3).

Hygiene and infection prevention measures were implemented according to the recommendations of the Commission for Hospital Hygiene and Infection Prevention (KRINKO) [13]. Between application of external fixation and definitive surgery, daily pin-site care was performed by trained nursing personnel. This included daily antiseptic cleaning with Octenisept (Schülke), application of sterile slit dressing pad and elastic bandaging of the fixator frame. All patients underwent routine screening for methicillin-resistant Staphylococcus aureus (MRSA) upon admission, with swabs obtained by trained nursing staff. Microbiological analysis followed current diagnostic standards, including up to 14 days of incubation.

Statistical analyses included chi-square testing to assess the association between fixator dwell time and colonisation rate. Results were considered significant at *p* < 0.05. Odds ratios with 95% confidence intervals were calculated. Associations between colonisation and patient-specific variables were examined univariately using chi-square tests for categorical variables and the Mann–Whitney U test for continuous data. Variables with significant univariate associations were subsequently included in a multivariate logistic regression model to identify independent predictors.

## Results

A total of 271 patients fulfilled the inclusion criteria and were analysed as a single consecutive cohort, comprising 153 men (56.5%) and 118 women (43.5%), with a median age of 56 years spanning a range from 18 to 99 years (Table 1).

**Table 1:** Baseline characteristics

Characteristic	Value
Age (Range)	18–99
BMI (Mean value ± SD)	28.5 ± 5.6 kg/m^2^
Sex (male)	153 (56.5%)
Sex (female)	118 (43.5%)
ASA 1	50 (18.5%)
ASA 2	157 (57.9%)
ASA 3	61 (22.5%)
ASA 4	3 (1.1%)
ASA ≥ 3 (dichotomised)	64 (23.6%)
Diabetes mellitus type 2	26 (9.6%)
Cardiovascular disease	118 (43.6%)
Nicotine abuse	71 (26.2%)

Definitive internal fixation was performed within ≤ 8 days in 151 patients (55.7%), whereas 120 patients (44.3%) underwent secondary fixation after > 8 days. Pin extraction occurred predominantly at the tibia (*n* = 214; 79.0%), while 48 cases (17.7%) involved the ulna in external fixations crossing the elbow joint, and isolated extractions were documented at the ilium (*n* = 2; 0.7%), radius (*n* = 4; 1.5%) and femur (*n* = 3; 1.1%). The mean duration of external fixation was 9.4 ± 4.8 days with a median of 8 days.

Sonication resulted in positive cultures for 88 of 271 patients (32.5%). In the ≤ 8-day group, 36 of 151 patients (23.8%) demonstrated microbial growth, whereas 52 of 120 patients (43.3%) in the > 8-day group showed colonisation, resulting in a statistically significant difference between the two strata (χ^2^ = 11.586; *p* = 0.001). Calculation of the odds ratio demonstrated a more than twofold increased likelihood of colonisation associated with external fixation times exceeding eight days (OR 2.44; 95% confidence interval (CI) 1.46–4.09), indicating a consistent shift in colonisation frequency across the duration threshold. When fixation duration was modelled as a continuous variable, each additional day of fixation was associated with increased odds of colonisation (OR 1.085 per day, 95% CI 1.011–1.165; *p* = 0.024). Inclusion of a quadratic term did not demonstrate significant non-linearity.

In total, 96 microorganisms were identified from all positive sonication cultures; in eight samples, two distinct microorganisms were detected (Table 2).

**Table 2:** Microorganisms identified in positive sonication samples (*n* = 96 isolates)

Pathogen	*n* (%)
Gram-positive bacteria	
Staphylococci	
*Staphylococcus epidermidis*	19 (19.8%)
*Staphylococcus capitis*	12 (12.5%)
*Staphylococcus aureus*	9 (9.4%)
*Staphylococcus haemolyticus*	9 (9.4%)
*Staphylococcus hominis*	8 (8.3%)
*Staphylococcus warneri*	3 (3.1%)
*Staphylococcus pettenkoferi*	2 (2.1%)
*Staphylococcus lugdunensis*	2 (2.1%)
*Staphylococcus saccharolyticus*	1 (1.0%)
*Staphylococcus pasteuri*	1 (1.0%)
Streptococci/Enterococci	
*Streptococcus dysgalactiae*	2 (2.1%)
*Streptococcus agalactiae*	1 (1.0%)
*Streptococcus viridans*	1 (1.0%)
*Enterococcus faecalis*	2 (2.1%)
Cutibacterium/Corynebacteriaceae	
*Cutibacterium acnes*	4 (4.2%)
*Corynebacterium striatum*	1 (1.0%)
*Corynebacterium pseudodiphtheriticum*	1 (1.0%)
*Corynebacterium aurimucosum*	1 (1.0%)
Bacillus spp. (Gram-positive, aerobic, Spore-forming)	
*Bacillus subtilis*	3 (3.1%)
*Bacillus cereus*	1 (1.0%)
*Bacillus licheniformis*	1 (1.0%)
*Bacillus simplex*	1 (1.0%)
Other gram-positive bacteria	
*Micrococcus luteus*	3 (3.1%)
*Kocuria rosea*	1 (1.0%)
*Globicatella sanguinis*	1 (1.0%)
Gram-negative bacteria	
*Pseudomonas stutzeri*	2 (2.1%)
*Pseudomonas oleovorans*	1 (1.0%)
*Escherichia coli*	1 (1.0%)
Fungi	
*Candida parapsilosis*	1 (1.0%)
*Candida albicans*	1 (1.0%)

Percentages refer to the total number of isolated microorganisms (*n* = 96). Multiple isolates per sample were possible.

Coagulase-negative staphylococci represented the predominant group and included Staphylococcus epidermidis (*n* = 19), Staphylococcus capitis (*n* = 12), Staphylococcus haemolyticus (*n* = 9) and Staphylococcus hominis (*n* = 8), while Staphylococcus aureus was isolated in nine cases, and additional isolates comprised Cutibacterium acnes (*n* = 4), Micrococcus luteus (*n* = 3), Pseudomonas stutzeri (*n* = 2) along with various corynebacteria, enterococci, streptococci and two Candida species. When microorganisms were grouped into four predefined categories (coagulase-negative staphylococci, Staphylococcus aureus, Candida spp. and other bacteria), no significant distribution difference between groups was detected (χ^2^ = 0.357; df = 3; *p* = 0.949), and coagulase-negative staphylococci remained the dominant group in both categories (≤ 8 days: 61.1%; > 8 days: 62.3%), without evidence of a shift in pathogen composition across duration strata.

Comorbidity profiling identified type 2 diabetes mellitus in 26 patients (9.6%), cardiovascular disease—including arterial hypertension, coronary artery disease and congestive heart failure—in 118 patients (43.6%), and active nicotine abuse in 71 cases (26.2%). The mean BMI was 28.5 ± 5.6 kg/m^2^. ASA classification showed the following distribution: ASA 1 18.5%, ASA 2 57.9%, ASA 3 22.5% and ASA 4 1.1%, with 64 patients (23.6%) classified as ASA ≥ 3. Univariate analyses demonstrated no significant associations between colonisation status and age, BMI, diabetes mellitus, nicotine abuse or cardiovascular disease. Correlation testing for BMI as a continuous variable showed no linear relationship with colonisation (Spearman *r* = 0.043; *p* = 0.484). Binary logistic regression including age, sex, BMI, ASA score, diabetes mellitus, nicotine abuse and cardiovascular disease identified male sex (*p* = 0.034; OR 1.904; 95% CI 1.050–3.455) and ASA ≥ 3 (*p* = 0.043; OR 1.588; 95% CI 1.014–2.487) as independent predictors of positive sonication, whereas all other variables showed no significant effect (age *p* = 0.767; BMI *p* = 0.905; diabetes mellitus *p* = 0.738; nicotine abuse *p* = 0.208; cardiovascular disease *p* = 0.803). When fixation duration was modelled as a continuous variable in multivariable logistic regression, a significant time-dependent association with colonisation was observed (OR 1.085 per day, 95% CI 1.011–1.165, *p* = 0.024), corresponding to an 8.5% increase in the odds of colonisation for each additional day of fixation. Inclusion of a quadratic term did not demonstrate significant non-linearity (*p* = 0.101).

Analysis of the microbial spectrum within comorbidity strata demonstrated no significant influence of patient characteristics on pathogen distribution. ASA classification (χ^2^ = 5.760; *p* = 0.124), diabetes mellitus (χ^2^ = 5.062; *p* = 0.167), nicotine abuse (χ^2^ = 1.609; *p* = 0.657), cardiovascular disease (χ^2^ = 0.078; *p* = 0.994) and elevated BMI ≥ 30 kg/m^2^ (χ^2^ = 1.410; *p* = 0.703) did not alter the distribution patterns of detected microorganisms, and across all examined strata, coagulase-negative staphylococci remained the dominant isolate group, whereas Staphylococcus aureus and Candida spp. appeared only sporadically and without identifiable distributional patterns within or across comorbidity categories.

## Discussion

**Fig. 4:**
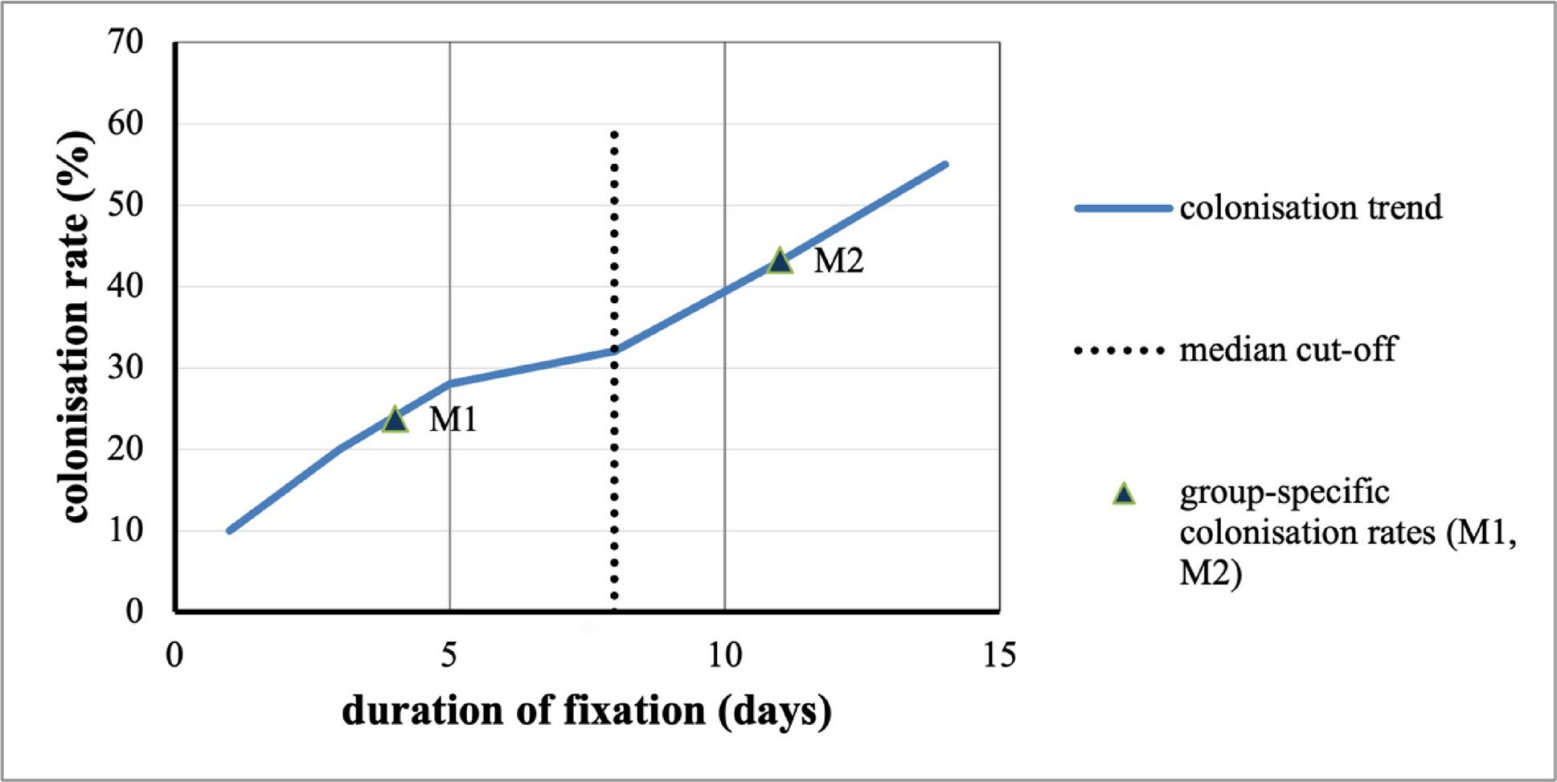
Time-dependent trend of microbial colonisation of external fixation pins. The curve depicts the proportion of positive sonication cultures from day 1 to day 14. The dotted vertical line marks the median cut-off at 8 days used for dichotomisation. M1 and M2 denote the group-specific colonisation rates for fixation durations ≤ 8 days (23.8%) and > 8 days (43.3%), respectively. The figure demonstrates a continuous increase in colonisation risk over time, with higher rates observed beyond the median fixation duration. The difference between fixation durations ≤ 8 days and > 8 days was statistically significant (χ^2^ = 11.586, *p* = 0.001). The curve illustrates the descriptive distribution across days; formal inference was based on logistic regression modelling

This study evaluated the association between the conversion time from external fixation to osteosynthesis and microbial colonisation of Schanz screws. Colonisation was detected in 23.8% of screws removed within eight days, rising to 43.3% when fixation exceeded eight days (OR 2.44; 95% CI 1.45–4.11; *p* = 0.001). The eight-day threshold reflects the median external fixation time of the cohort and does not represent a biological cut-off. Across the observed spectrum from day 1 to day 14, colonisation demonstrated a continuous increase with maximum values from day 11 onward (Fig. 4). In addition to this dichotomized analysis, fixation duration was modelled as a continuous variable in multivariable logistic regression. Each additional day of fixation was associated with an 8.5% increase in the odds of colonisation (OR 1.085 per day), without evidence of significant non-linearity. These findings support a gradual time-dependent increase rather than the presence of a discrete temporal threshold.

The median fixation time in Group 1 (≤ 8 days) was 6 days (interquartile range [IQR] 5–7), whereas Group 2 (> 8 days) showed a median fixation time of 12 days (interquartile range [IQR] 10–13). These findings indicate a time-dependent rise in colonisation risk corresponding to the clinically established interval for conversion to definitive osteosynthesis described in the damage control concept [14, 15].

Both experimental studies and clinical investigations have explored the potential influence of temporary external fixation on outcomes during subsequent definitive osteosynthesis in multi-stage treatment concepts. For example, Clasper et al. demonstrated in their recent study bacterial migration several centimetres from the insertion site of Schanz screws into the medullary canal and reported septic arthritis, abscess formation and osteomyelitis during subsequent intramedullary procedures [11]. Moreover, Fritz et al. likewise reported in their clinical investigations that a longer time interval between temporary external fixation and definitive osteosynthesis might be associated with an increased infection risk. In their retrospective analysis of multi-stage treatment of distal radius fractures, a significantly higher infection rate (9.4%) was documented when conversion from external to internal fixation was delayed beyond 14 days [16].

Yeramosu et al. and Bunzel et al. confirmed these observations in their studies on staged management of lower-leg fractures, each demonstrating increased infection rates with extended periods of external fixation [17, 18].

With regard to the microbial profile of colonisation, our present analysis demonstrated that no significant differences in the microbial spectrum were detected between the two groups (χ^2^ = 0.357; *p* = 0.949). Coagulase-negative staphylococci predominated in both groups (approximately 62%). Staphylococcus aureus and Candida spp. were detected only sporadically. Thus, longer external fixation time increased colonisation probability without altering pathogen composition. Accordingly, Knabl et al. conducted a prospective cohort study to assess microbial colonisation of explanted osteosynthesis material in patients without clinical signs of infection. All implants were processed by sonication, followed by culture and polymerase chain reaction (PCR) of the sonication fluid, and selected samples were examined using scanning electron microscopy. Microbial colonisation was detected in 56.1% of implants, with coagulase-negative staphylococci accounting for 68.1% of isolates. Culture yielded higher detection rates than PCR (50.9% vs. 21.1%), and biofilm-like structures were confirmed microscopically in two-thirds of microbiologically positive samples. In contrast to the present findings, comparative analyses of external fixation systems reported no significant association between fixation duration and microbial colonisation [19].

In addition, several patient-related variables were examined in our study to assess their potential influence on Schanz screw colonisation. When comparing the two groups, an ASA score ≥ 3 was associated with higher colonisation rates of the Schanz screws (OR 1.59; 95% CI 1.01–2.49; *p* = 0.043), whereas other comorbidities, including type 2 diabetes mellitus, cardiovascular disease, BMI and nicotine abuse, showed no significant association with colonisation in either group. Male sex was associated with higher colonisation rates (OR 1.90; *p* = 0.03), without a clear biological mechanism. None of the examined comorbidities influenced the microbial spectrum.

These findings correspond with previously published clinical data indicating an elevated infection risk with increasing ASA class [20, 21]. The ASA score, as a non-specific marker of systemic disease burden, may indicate increased susceptibility without representing a direct causal factor. The narrow BMI range and overall favourable comorbidity profile of the cohort may explain the absence of further associations. The non-significant trend in smokers aligns with previously described effects of nicotine on wound healing [22–24].

Implant surface characteristics are known to influence bone–implant interaction and mechanical fixation, particularly for titanium alloys and surface-modified implants. Experimental studies have demonstrated improved osseointegration for coated titanium surfaces under load-bearing conditions [25, 26]. To minimise confounding by material-related effects, all Schanz screws used in the present study were of identical composition (TiAl6Nb7), allowing fixation duration and patient-related factors to be analysed under uniform implant conditions.

When interpreting these results, aspects of the study protocol as well as several methodological limitations must be taken into account. First, open fractures were excluded to avoid confounding by trauma-related contamination and to ensure that detected microorganisms reflected colonisation acquired during implant retention. Similarly, patients initially treated at external centres were excluded to maintain uniform surgical standards and microbiological processing, thereby reducing heterogeneity and strengthening internal validity. Second, despite the use of standardised removal techniques, retrograde explantation inherently carries a risk of contamination by skin flora, even though strict sterile precautions were applied prior to pin removal. Third, sonication offers high sensitivity (77–79%) and specificity (96–99%) for biofilm-associated bacteria [27, 28] but may also detect clinically irrelevant microorganisms [29, 30]. The study design did not include baseline swabs, so preoperative subclinical colonisation cannot be excluded. Furthermore, most screws originated from tibial fixations. Although no location-dependent differences in colonisation rates were observed in our analysis, it must be acknowledged that the composition of the resident skin microbiota varies by anatomical region, particularly given the lower prevalence of Cutibacterium spp. in the lower extremity [31–33]. The predominance of tibial screws therefore introduces a potential bias, as the microbial spectrum identified in this cohort may not fully reflect colonisation patterns at other fixation sites. Finally, while sonication reliably detects microbial presence, it does not differentiate between contamination, colonisation, and infection, requiring clinical correlation before therapeutic conclusions are drawn. In the context of this study, colonisation refers to the microbiological detection of viable organisms on explanted screw tips in the absence of clinical signs of infection. Fracture-related infection (FRI), in contrast, is a clinically defined entity requiring confirmatory criteria such as purulence, sinus tract formation, pathogen isolation from deep tissue samples, or histopathological evidence [34]. Sonication-positive findings alone do not fulfil these diagnostic criteria and must therefore not be interpreted as evidence of clinically manifest infection. Future research should prospectively evaluate whether sonication-positive colonisation of temporary external fixations is associated with subsequent fracture-related infection (FRI) after definitive osteosynthesis. Such studies should apply standardized FRI diagnostic criteria and longitudinal follow-up in order to determine whether colonisation represents a predictive marker, a coincidental microbiological finding, or a clinically irrelevant phenomenon. Only through correlation with validated infection endpoints can the temporal dynamics observed in the present study be translated into clinically meaningful risk stratification.

## Conclusions

External fixation remains a widely used and indispensable component of staged fracture management in trauma care. Given the large number of patients treated with temporary external fixators, even subclinical microbial colonisation may warrant further investigation regarding its potential clinical relevance. The present findings indicate that prolonged fixation duration and higher systemic disease burden, reflected by an ASA score ≥ 3, are associated with increased microbial colonisation of Schanz screw tips. This study did not evaluate clinical infection outcomes (including FRI); therefore, the findings should not be used to derive therapeutic recommendations or intervention thresholds. Further analyses will focus on the clinical implications of microbial colonisation in this patient population.

## Data Availability

The datasets of the current study are not publicly available. Data will be available upon request to the first author, TK.

## Abbreviations

ASA: American Society of Anesthesiologists
BMI: Body mass index
CFU: Colony–forming units
CI: Confidence interval
ISS: Injury Severity Score
IQR: Interquartile range
MRSA: Methicillin-resistant *Staphylococcus aureus*
OR: Odds ratio
PCR: Polymerase chain reaction
